# Shifting Cancer Landscapes in Korea: Divergent Incidence Trajectories, Sex-Specific Burdens, and Projected Case Counts for 24 Major Cancer Types from 1999 to 2023, with Forecasts to 2030

**DOI:** 10.3390/curroncol33080444

**Published:** 2026-07-24

**Authors:** Hyeran Jung, Minsun Jung

**Affiliations:** Department of Pathology, Yonsei University College of Medicine, Seoul 03722, Republic of Korea; phdgrace@yuhs.ac

**Keywords:** neoplasms, incidence, registries, Republic of Korea, sex distribution, forecasting, age distribution, population surveillance, epidemiologic studies

## Abstract

Background/Objectives: Korea has experienced rapid and heterogeneous shifts in cancer epidemiology since 1999. We aimed to characterize divergent incidence trajectories across the 24 major cancer types, quantify sex-specific burdens, and project case counts through 2030. Methods: Annual incidence data—including new case counts and age-standardized incidence rates (ASIRs) per 100,000 (2020 Korean standard population) stratified by sex—were extracted from the KCCR via the Korean Statistical Information Service (KOSIS) for 1999–2023. Annual percent change (APC) was estimated by log-linear regression with 95% confidence intervals (CIs). Holt–Winters damped exponential smoothing generated 2024–2030 projections with 95% prediction intervals (PIs). Changes in case ascertainment and coding over the study period were considered in interpretation. Results: Total incidence increased from 101,854 (1999) to 288,613 cases (2023), a 183.4% increase. Overall ASIR rose from 402.7 to 522.9 per 100,000. Of the 24 cancer types, 15 showed statistically significant increasing ASIR trends, 6 showed significant decreasing trends, and 3 showed no significant change. The highest-APC cancers were thyroid (+7.56%), prostate (+6.98%), testis (+5.39%), breast (+5.03%), and corpus uteri (+5.03%; all *p* < 0.001). The largest significant declines occurred in cervix uteri (−3.81%), larynx (−3.18%), liver (−2.90%), and stomach (−2.20%; all *p* < 0.001). Female ASIR increased from 294.7 to 488.9 per 100,000 (+65.9%); male ASIR increased from 573.3 to 587.0 per 100,000 (+2.4%). Total cancer incidence is projected to reach 307,091 (95% PI: 281,200–332,983) in 2026 and 330,213 (95% PI: 290,663–369,763) by 2030, with the steepest projected relative growth for prostate (+48.9%), kidney (+33.7%), and breast (+31.3%) cancers. Conclusions: Korean cancer epidemiology is undergoing a pronounced transition from infection-related toward metabolic, hormonal, and aging-related malignancies, with a marked and widening sex-specific divergence. Cancer-type-resolved projections through 2030 provide an evidence base for strategic capacity planning in Korean oncology.

## Simple Summary

Korea has undergone a fundamental restructuring of its cancer burden over the past quarter-century. Using 25 years of nationwide cancer registry data from the Korea Central Cancer Registry (KCCR) for the 24 major cancer types defined in national reporting (1999–2023), this study characterizes divergent age-standardized incidence rate (ASIR) trajectories, quantifies sex-specific burdens, and projects case counts through 2030 using Holt–Winters exponential smoothing. The fastest-growing cancer types by annual percent change (APC) were thyroid (+7.56%/year), prostate (+6.98%), testis (+5.39%), breast (+5.03%), and corpus uteri (+5.03%). Conversely, cervix uteri (−3.81%), larynx (−3.18%), liver (−2.90%), and stomach (−2.20%) cancers declined significantly. Female ASIR rose by 65.9%, far exceeding the 2.4% increase in male ASIR, driven predominantly by thyroid, breast, and corpus uteri cancers. Total cancer incidence is projected to reach approximately 330,000 cases by 2030. Because case ascertainment and diagnostic practices have also improved over the study period, part of the apparent increase reflects changes in detection and registration; these cancer-type-resolved findings nonetheless provide a quantitative foundation for national cancer control planning in Korea.

## 1. Introduction

Cancer is the leading cause of mortality in the Republic of Korea, accounting for approximately 27% of all deaths in recent years and imposing a profound burden on the healthcare system and society [1,2]. Over the past quarter-century, Korea has undergone one of the most rapid and multifaceted epidemiological transitions in cancer documented among high-income East Asian nations, driven by the convergence of demographic aging, behavioral westernization, expanded population-based screening programs, and a declining prevalence of infection-related risk factors [3,4].

The KCCR, administered under the Korea Central Cancer Registry program, provides population-level cancer incidence data with completeness estimated at greater than 97%, offering a high-quality platform for epidemiological analysis [1,5]. Annual reports consistently document a sustained rise in overall cancer incidence, from approximately 102,000 new cases in 1999 to more than 288,000 in 2023, an approximately 183% increase [1,4]. However, this aggregate trajectory conceals highly heterogeneous cancer-type-specific trends: thyroid cancer underwent a greater-than-tenfold increase in case count, prostate cancer rose more than fifteenfold, and stomach cancer declined by more than 40% in age-standardized rate [1,6,7]. Importantly, the magnitude of some of these increases must be interpreted in the context of parallel improvements in diagnostic technology, case ascertainment, and registry coding practices over the same period, which can inflate apparent incidence for screen-detected and pathologically reclassified cancers [8,9].

A particularly important but underemphasized dimension of Korea’s cancer transition is the sex-specific divergence in incidence rates. Female ASIR rose by an estimated 65.9% between 1999 and 2023, far exceeding the modest 2.4% increase observed in males. This divergence is attributable to the disproportionate impact of thyroid, breast, and corpus uteri cancers in women—malignancies characterized by strong hormonal, metabolic, and lifestyle-related drivers—and has direct implications for the design of cancer screening programs and specialist workforce planning [9,10].

Despite the scientific importance and policy relevance of these trends, existing Korean cancer surveillance literature has largely focused on shorter observation periods, a restricted set of cancer types, or aggregate statistics without cancer-type-resolved projections [6,7,9,11,12]. Three specific gaps remain. First, no prior study has simultaneously characterized ASIR trajectories, APC, and sex-disaggregated absolute burden for all 24 major cancer types across the complete 25-year national series (1999–2023). Second, most projection studies address only total incidence or a handful of common cancers, leaving the fastest-changing malignancies (e.g., prostate, testis, thyroid) and the emerging hematological cancers without validated multi-horizon forecasts. Third, the interpretive influence of secular changes in data recording and case ascertainment has rarely been made explicit alongside the trend estimates. Addressing these gaps is essential for translating surveillance data into actionable capacity planning.

The present study addresses these gaps with the following objectives: (1) to characterize individual ASIR trajectories and APC with 95% CIs for all 24 major cancer types; (2) to quantify sex-specific absolute and relative ASIR changes; and (3) to generate Holt–Winters incidence projections for 2024–2030 for all 24 cancer types and the national total, while explicitly considering the contribution of secular changes in diagnosis and registration to observed trends.

## 2. Materials and Methods

### 2.1. Data Source and Study Period

Annual cancer incidence data (1999–2023) were obtained from the KCCR, administered by the Ministry of Health and Welfare of the Republic of Korea, and publicly accessible through KOSIS (https://kosis.kr (accessed on 25 May 2026); Table ID: DT_117N_A00025). The KCCR is a nationwide, population-based registry established in 1980 that has reported comprehensive national statistics since 1999. Incident cases are ascertained through mandatory reporting from all medical institutions and are supplemented by record linkage with the National Health Insurance Service claims database, the Korea Central Cancer Registry hospital network, and national death certificate data, yielding registry completeness estimated at 97.4% for 2023 [1,5]. Cancers are coded according to the International Classification of Diseases for Oncology, Third Edition (ICD-O-3), and converted to the corresponding ICD-10 topography categories used in national reporting. In December 2025, the 2023 cancer statistics were officially released with concurrent retrospective revision of all 1999–2022 data; this study uses the fully revised dataset to maximize internal consistency across the series.

For each of the 24 major cancer types defined in the KCCR national reporting framework (Table 1) and for all cancers combined, the following variables were extracted by sex (total, male, and female): annual new case count (N); crude incidence rate (per 100,000 population); and ASIR (per 100,000). ASIRs were standardized to the 2020 Korean mid-year resident-registration population using the direct method, in which age-specific incidence rates (in 5-year age strata) were weighted by the corresponding standard-population age structure and summed. Sex-specific ASIRs were standardized to the sex-specific standard population. All ASIR values used in this study are those officially published by the KCCR; no re-computation from raw microdata was performed.

### 2.2. Data Quality and Comparability over Time

Because the study spans 25 years, changes in diagnostic technology, screening intensity, and coding conventions can affect the comparability of incidence counts across time. Three sources of secular artifacts were considered explicitly. First, the diffusion of high-resolution imaging (notably thyroid ultrasonography and cross-sectional imaging) and of organized and opportunistic screening increased the detection of early-stage and indolent disease, contributing to apparent incidence increases that do not necessarily reflect rising true disease occurrence [8,11]. Second, revisions in histopathological classification and improvements in subtype-specific coding shifted cases between diagnostic categories and improved their capture over the series. Third, registry completeness itself improved over time, from early nationwide reporting toward the >97% completeness attained in recent years [1,5]. These factors are quantitatively inseparable from genuine epidemiological change in an aggregate registry analysis; we therefore interpret large relative increases conservatively and address their likely composition in the Discussion.

### 2.3. Annual Percent Change (APC)

For each of the 24 cancer types, APC was estimated by fitting a log-linear regression model to the ASIR time series: ln(ASIR_t_) = α + β × t. The APC was computed as APC (%) = (e^β^ − 1) × 100 [13]. The 95% CI for APC was derived from the standard error of β. Statistical significance was assessed by two-sided *t*-tests (α = 0.05). This model assumes an approximately constant proportional (geometric) rate of change over the observation period, independence of the annual observations conditional on the trend, and approximately log-normal errors; these assumptions are reasonable for smoothly evolving registry series but are weaker where abrupt inflections occur (e.g., the thyroid screening reversal and the 2020 pandemic dip), which we note where relevant. All 24 cancer types had complete 25-year ASIR series and were included in the APC analysis.

### 2.4. Holt–Winters Forecasting

Cancer-specific incidence projections for 2024–2030 were generated using Holt–Winters damped exponential smoothing with an additive trend component [14,15], implemented in Python 3.12 (statsmodels 0.14 ExponentialSmoothing). No seasonal component was specified because the data are annual. Model parameters (α, β, φ) were optimized by maximum likelihood estimation. Forecast uncertainty was quantified as 95% PIs using PI = FC ± 1.96 × σ_resid_ × √h, where σ_resid_ is the in-sample residual standard deviation, and h is the forecast horizon in years [16]. The method assumes that the level and (damped) trend structure estimated over 1999–2023 remain a reasonable description of the near-term future and that residuals are approximately homoscedastic; the damping parameter φ attenuates trend extrapolation to guard against implausible long-horizon divergence. Because these assumptions cannot accommodate future structural breaks (new guidelines, novel interventions, or pandemics), projections are presented as scenario estimates rather than deterministic predictions, and the widening PIs with the horizon reflect this uncertainty.

### 2.5. Software and Reporting

All analyses were performed using Python 3.12 (NumPy 1.26, pandas 2.2, SciPy 1.12, statsmodels 0.14, and Matplotlib 3.8). The study adheres to the STROBE reporting guideline for observational studies.

### 2.6. Ethical Statement

This study used exclusively publicly available, fully de-identified aggregate cancer registry statistics. No individual-level patient data were accessed or analyzed. In accordance with applicable institutional guidelines, ethical review and approval were waived by the Institutional Review Board of Yonsei University (IRB No. 4-2026-0352). Informed consent was waived because the study used de-identified aggregate national statistics and did not involve direct contact with human participants.

## 3. Results

### 3.1. Divergent Trajectories and Trend Classification

**Figure 1:**
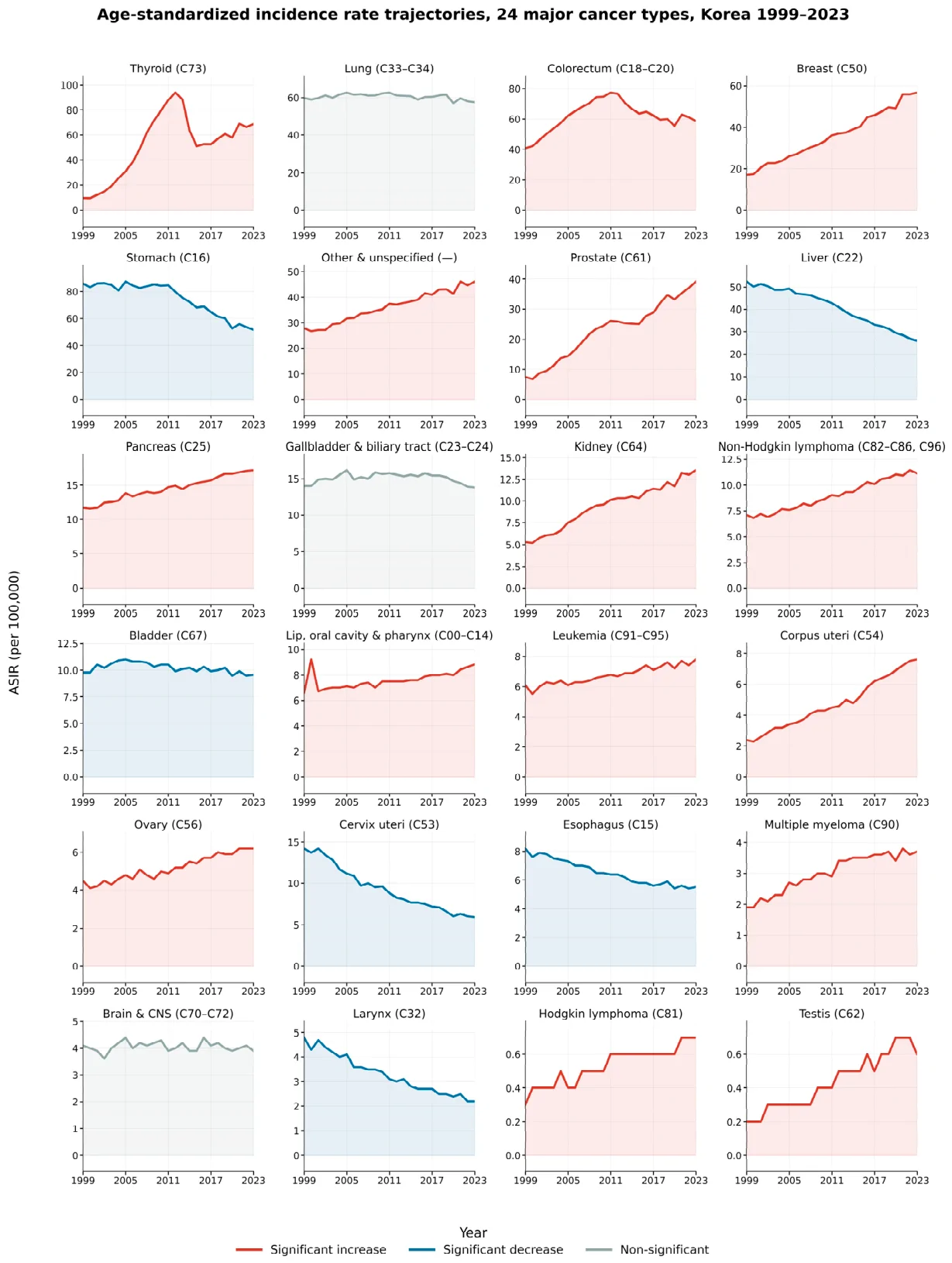
Age-standardized incidence rate (ASIR; per 100,000) trajectories for all 24 major cancer types in Korea, 1999–2023, shown as small multiples. Line color denotes the direction and statistical significance of the log-linear trend: red indicates a statistically significant increasing trend, blue a statistically significant decreasing trend, and grey a non-significant trend (*p* ≥ 0.05). Panels are ordered by 2023 case count. Note that the vertical scale differs across panels.

Figure 1 shows the ASIR trajectory of every cancer type; the corresponding APC estimates with 95% CIs are reported in Table 1. Classified by the significance of the log-linear trend, 15 cancer types increased significantly, 6 decreased significantly, and 3 types—lung, gallbladder and biliary tract, and brain/CNS—showed no significant change. Two features dominate the panel. First, the strongest sustained increases clustered in screening-detectable and hormone-related cancers, led by thyroid and prostate, whereas the steepest declines occurred in infection- and tobacco-related cancers such as stomach, liver, cervix uteri, and larynx. Second, a transient dip in 2020, visible across many panels, reflects the pandemic-related interruption of screening, with incidence recovering toward pre-pandemic trajectories by 2021–2022. As noted in Section 2.2, part of the largest increases—most notably thyroid—reflects improved detection and ascertainment rather than a true rise in disease occurrence alone.

### 3.2. Reordering of the Cancer Burden, 1999–2023

**Figure 2:**
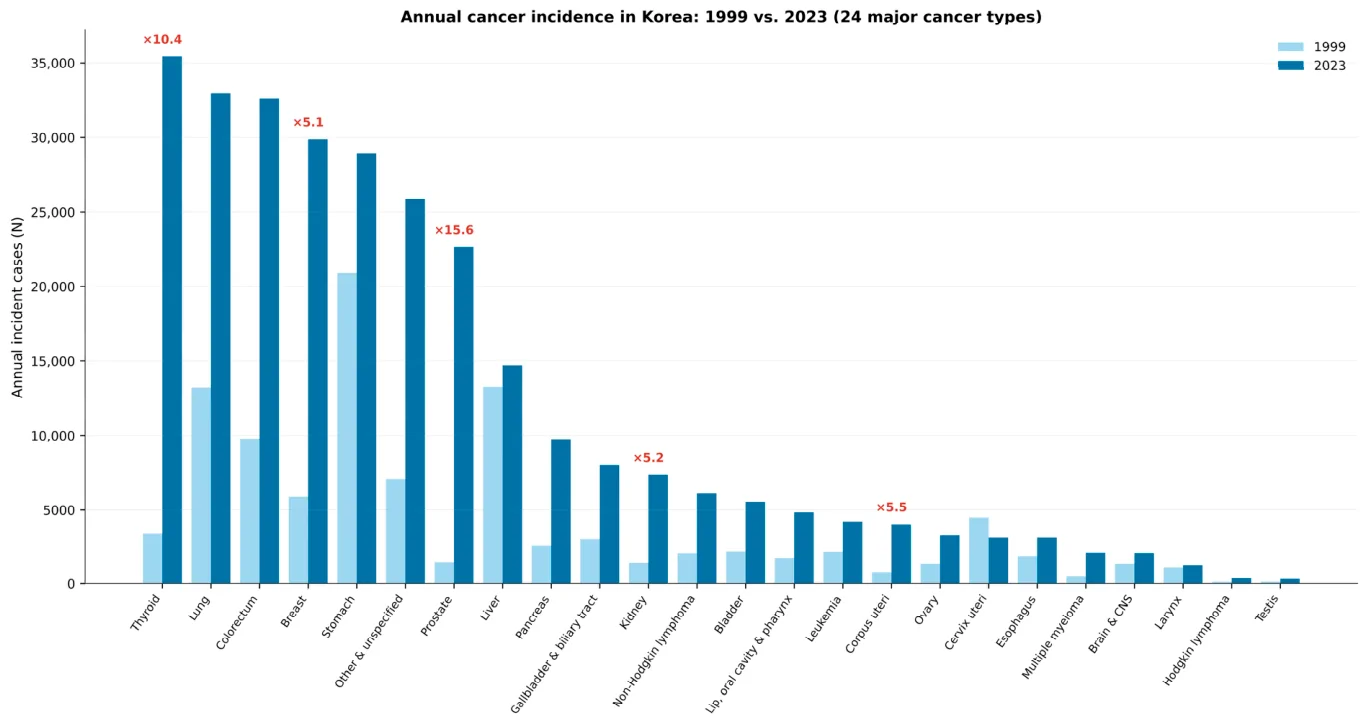
Grouped bar chart comparing annual cancer incidence (new case counts) in 1999 (light blue) and 2023 (dark blue) for all 24 major cancer types. Fold-change annotations (×n) are shown for the five cancer types with the greatest relative increase in case count. Cancer types are arranged in decreasing order of 2023 case count.

**Figure 3:**
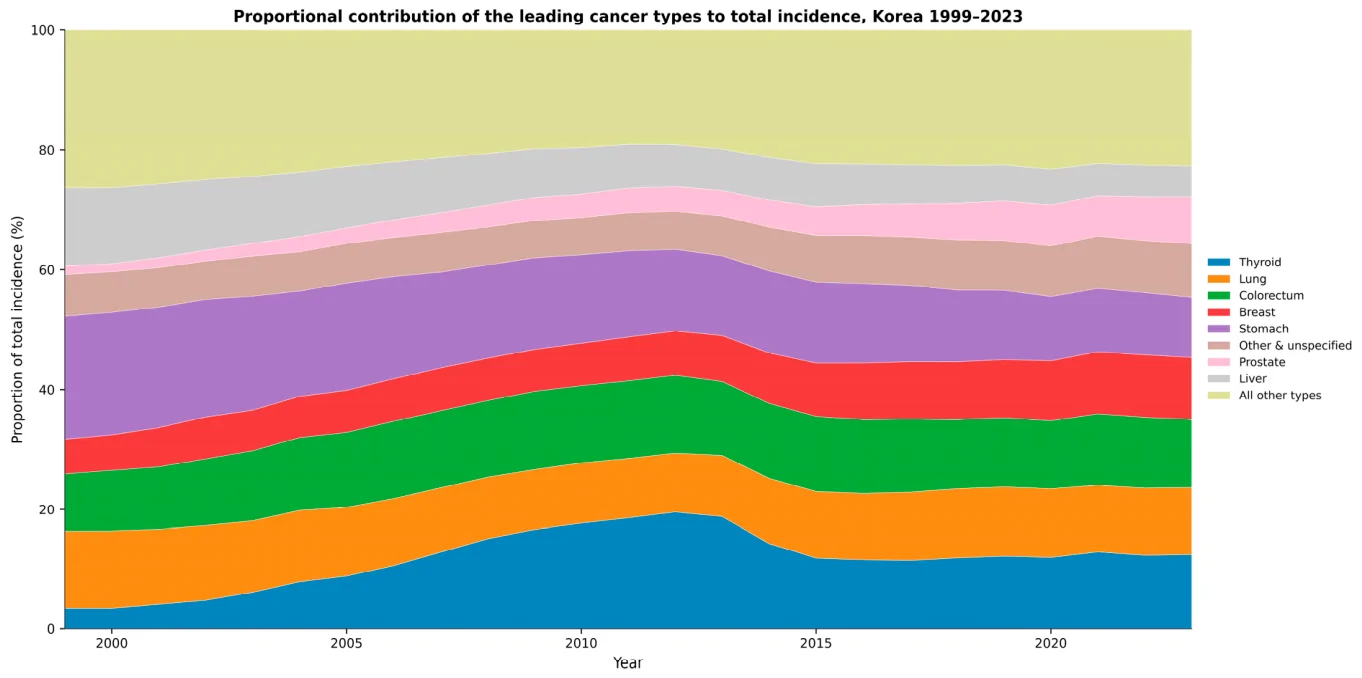
Stacked area chart showing the proportional contribution (%) of the eight leading cancer types (by 2023 case count) to total annual cancer incidence in Korea, 1999–2023. The progressive enlargement of the thyroid, breast, and prostate shares and the sustained contraction of stomach cancer’s share illustrate the structural transformation of Korea’s cancer burden over 25 years.

The composition of the national cancer burden was substantially reordered over the study period (Figure 2 and Figure 3; case counts in Table 1). In 1999 the leading cancers were infection-related, with stomach and liver foremost; by 2023 the burden was headed by thyroid, lung, colorectum, breast, and stomach, with prostate close behind. Relative growth was greatest for prostate and thyroid, each rising by roughly an order of magnitude, followed by corpus uteri, kidney, and breast (fold changes annotated in Figure 2). This reordering is mirrored in the proportional composition of incidence (Figure 3): the combined share of thyroid, breast, and prostate cancers expanded markedly while the share of stomach cancer approximately halved, illustrating the shift from infection-related toward screening-detectable, hormone-related, and lifestyle-related malignancies.

### 3.3. Sex-Specific Divergence

**Figure 4:**
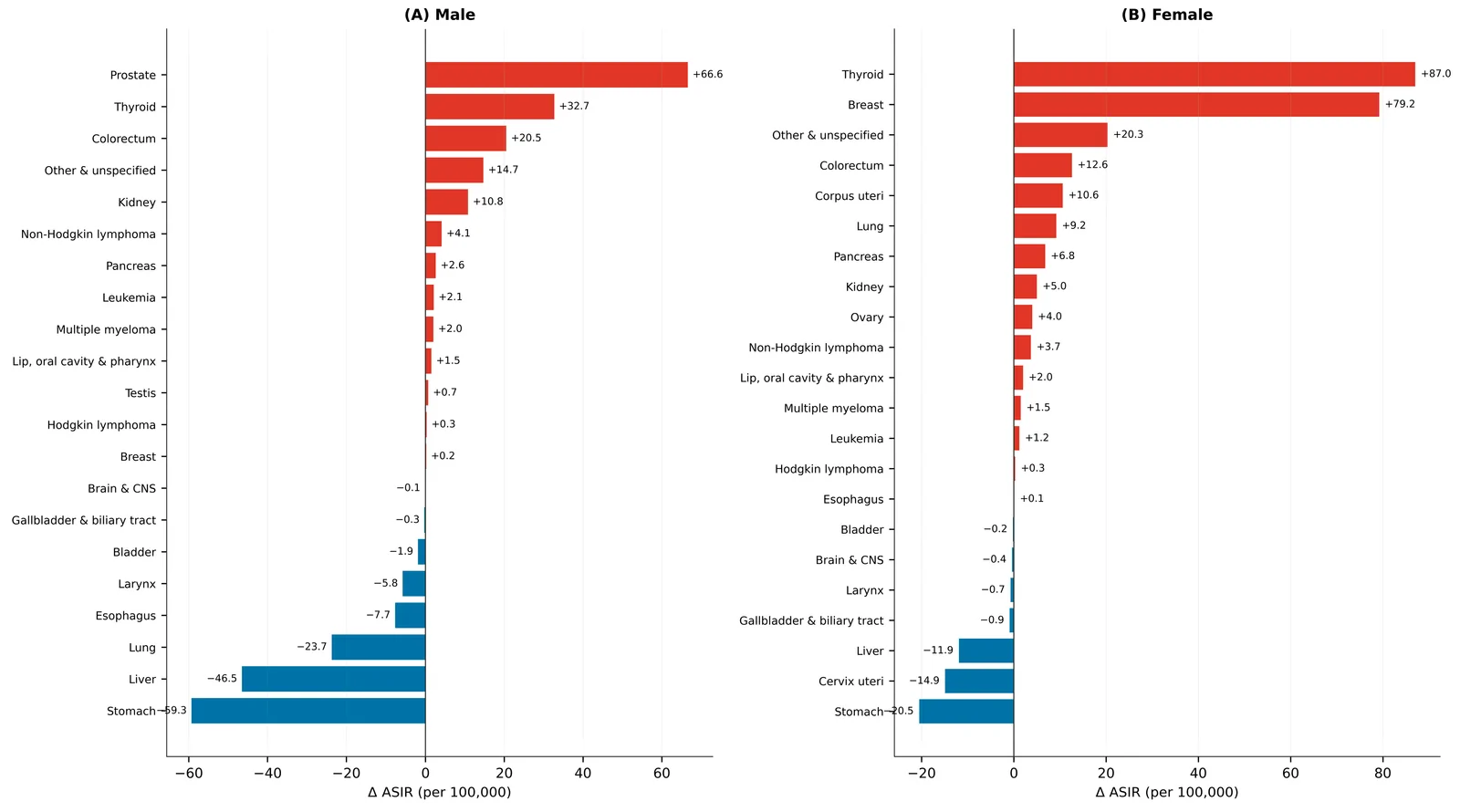
Sex-specific diverging bar charts of the absolute change in age-standardized incidence rate (ASIR; per 100,000) from 1999 to 2023: (**A**) male ASIR change; (**B**) female ASIR change. Values on bars indicate the magnitude of change per 100,000. Red bars: ASIR increase; blue bars: ASIR decrease. Cancer types are ordered by the magnitude of ASIR change. The horizontal scale differs between panels, reflecting the substantially greater magnitude of female ASIR changes.

The transition was strongly sex-dependent (Figure 4). Overall, female ASIR rose by about two-thirds (from 294.7 to 488.9 per 100,000; +65.9%), whereas male ASIR was essentially unchanged (from 573.3 to 587.0 per 100,000; +2.4%). In women, the divergence was driven overwhelmingly by thyroid and breast cancers, with a smaller contribution from corpus uteri; in men, pronounced increases in prostate and thyroid cancers were largely offset by steep declines in stomach, liver, and lung cancers, yielding a near-flat net trajectory. The magnitude of change was considerably greater in women than in men (note the differing horizontal scales in Figure 4). Sex-specific ASIRs are standardized to the corresponding sex-specific standard population and therefore differ from the total-population ASIRs in Table 1 for sex-predominant cancers.

### 3.4. Annual Percent Change and Projections to 2030

**Figure 5:**
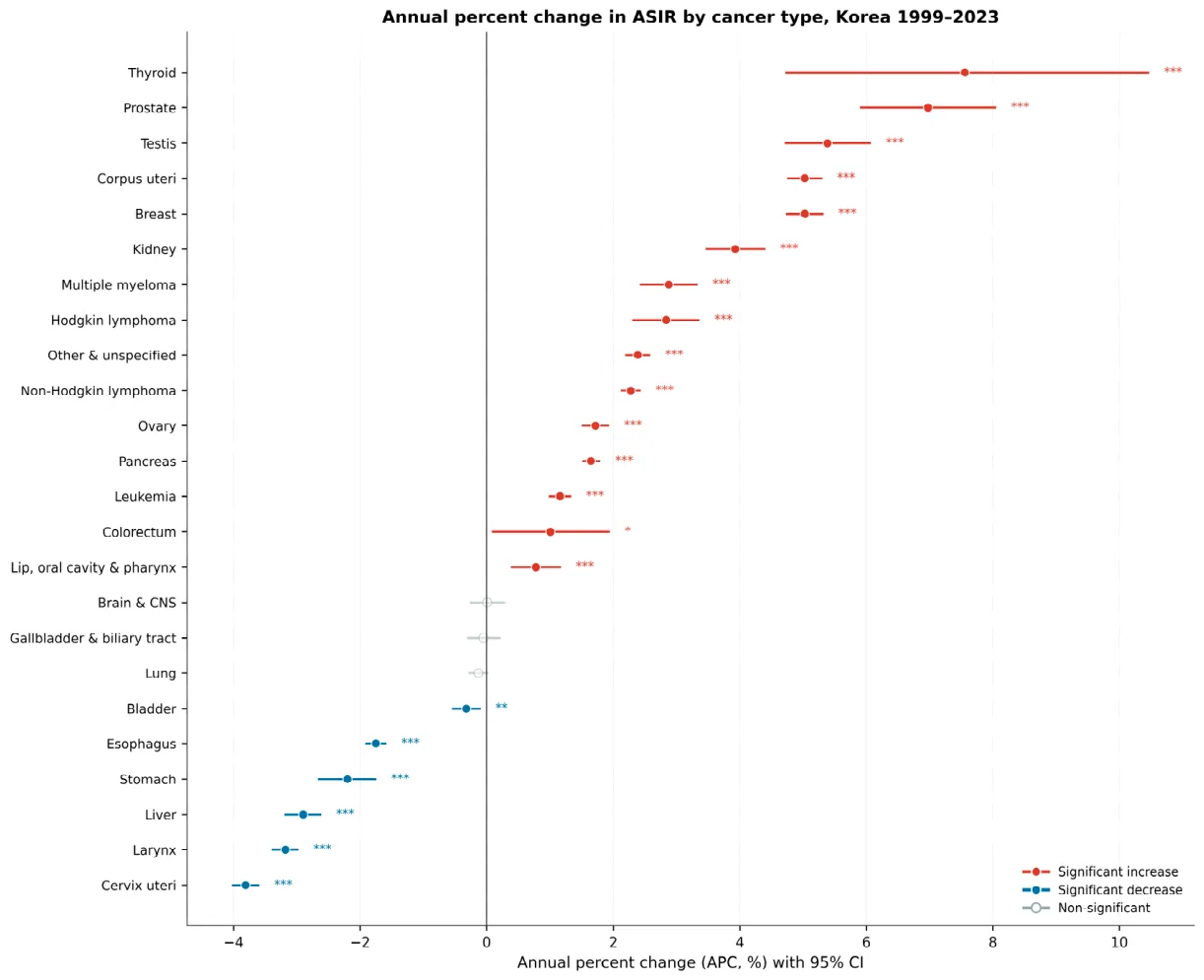
Lollipop chart of annual percent change (APC) in age-standardized incidence rates with 95% confidence intervals for all 24 major cancer types in Korea (1999–2023), estimated by log-linear regression. Filled markers and opaque lines indicate statistically significant trends (*p* < 0.05); open markers and faded lines indicate non-significant trends. Red: increasing; blue: decreasing. Significance: *** *p* < 0.001; ** *p* < 0.01; * *p* < 0.05.

**Figure 6:**
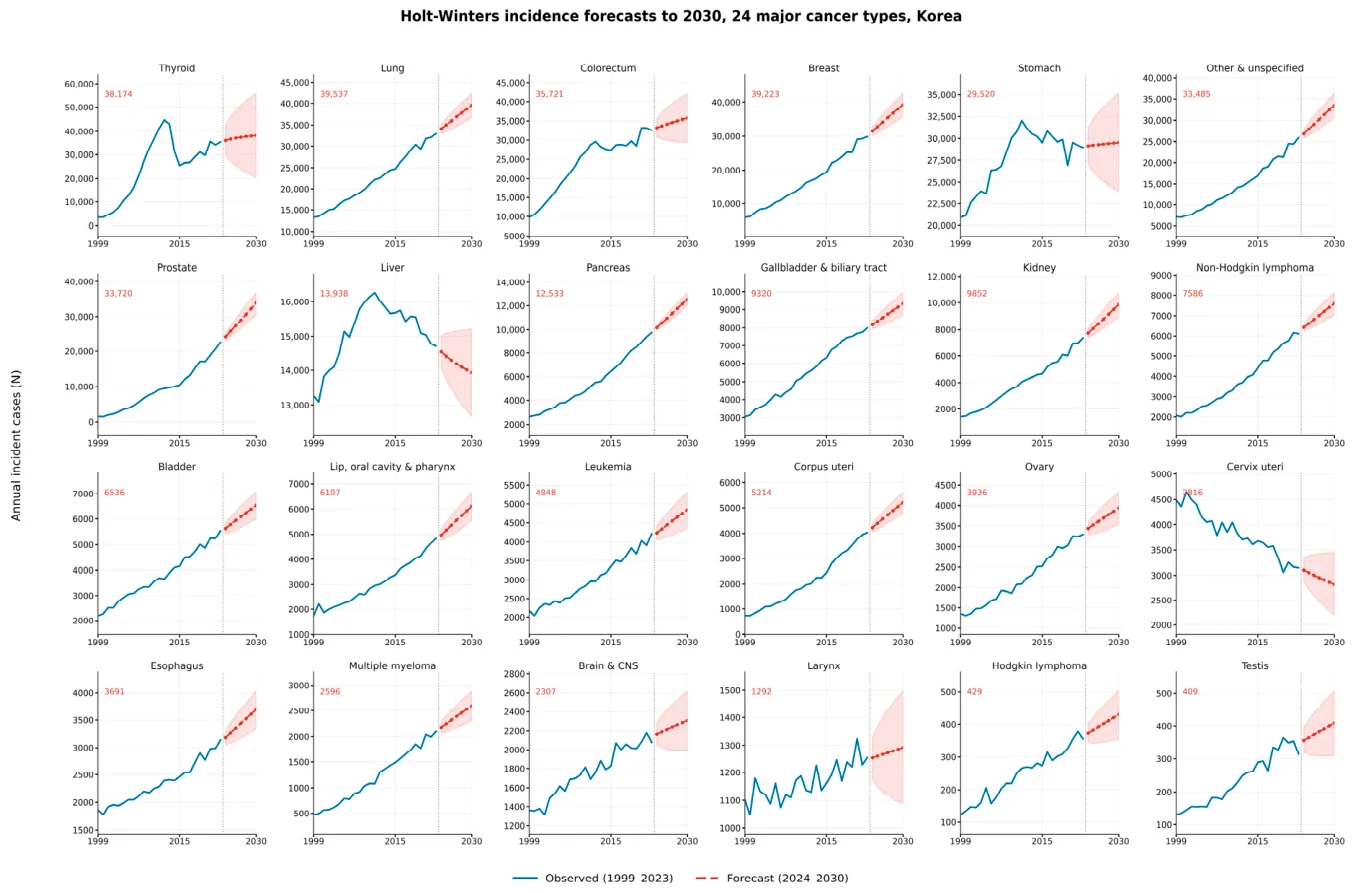
Holt–Winters damped exponential smoothing forecasts (2024–2030) for all 24 major cancer types, presented as small multiples. Blue lines represent observed incidence (1999–2023); red dashed lines with markers represent projected incidence (2024–2030); shaded areas indicate 95% prediction intervals. The projected 2030 case count is annotated in each panel. The vertical dotted line separates observed from projected data.

Figure 5 ranks all cancer types by APC (values with 95% CIs in Table 1). The fastest significant increases were in thyroid, prostate, and testicular cancers, followed by breast and corpus uteri cancers and a consistent group of hematological malignancies (multiple myeloma and Hodgkin and non-Hodgkin lymphoma); the steepest significant declines were in cervix uteri, larynx, liver, and stomach cancers. Holt–Winters projections to 2030 are shown in Figure 6, with numerical detail in Table 2. Total incidence is projected to reach approximately 330,000 cases by 2030 (95% PI: 290,663–369,763). The steepest relative growth is projected for prostate, kidney, and breast cancers, whereas thyroid incidence is projected to plateau—consistent with post-overdiagnosis stabilization [8]—and liver and cervical cancers are projected to continue declining. Prediction intervals widen with the forecast horizon, underscoring the scenario nature of the projections.

## 4. Discussion

This study provides a cancer-type-resolved epidemiological analysis of all 24 major cancer types. It spans the complete 25-year national registry period (1999–2023). The analysis integrates sex-disaggregated burden quantification, log-linear APC estimation with significance testing, and validated Holt–Winters projections through 2030. The findings document a profound and ongoing epidemiological transition. This transition has direct implications for oncological care planning, specialist workforce development, and cancer screening program design in Korea. These trends must nonetheless be interpreted with caution. A portion of the observed change reflects secular improvements in diagnosis and registration rather than true shifts in disease occurrence (Section 2.2).

The most striking finding is the magnitude of the sex-specific divergence in incidence trends (quantified in the Results and Figure 4). Over the study period, female incidence rose by roughly two-thirds. Male incidence, by contrast, remained essentially flat. This divergence was driven predominantly by thyroid and breast cancers in women, with a smaller contribution from corpus uteri cancer. Several factors plausibly explain the disproportionate female burden. These include opportunistic thyroid ultrasonography [8,17], the westernization of reproductive and lifestyle risk factors affecting breast and uterine cancers [10], and the expansion of organized mammography screening [11]. Ecological analyses in other populations have similarly linked rising uterine and hormone-related cancer incidence to metabolic and dietary transitions accompanying socioeconomic development [18]. This pattern is consistent with the drivers inferred here. Together, these findings argue for prioritizing sex-specific prevention strategies and female-targeted oncological resource allocation.

In men, the near-flat net trajectory masks two opposing forces. Substantial increases in prostate and thyroid cancers were largely offset by pronounced declines in stomach, liver, and lung cancers. Stomach and liver cancers are strongly linked to Helicobacter pylori and hepatitis B virus infection, respectively. Their steep retreat epitomizes the decline of infection-associated cancers. This decline follows improved sanitation, H. pylori eradication, universal hepatitis B vaccination, and antiviral therapy. Larynx and esophageal cancers, both strongly tobacco- and alcohol-related, declined in parallel. These declines are consistent with the long-term reduction in male smoking prevalence in Korea. Collectively, these shifts illustrate how the male burden is being redistributed from infection- and tobacco-related cancers toward screening- and lifestyle-associated types.

Prostate cancer shows the steepest projected relative growth of any type through 2030 (Results, Table 2). Relative to Western nations, Korea remains at an early-intermediate stage of prostate-specific antigen (PSA) screening diffusion, with a still-low national testing rate among older men [19]. Both screening intensity and population aging are expected to increase. This trajectory is consistent in direction with prior Korean-specific projection studies [19,20]. It underscores the need for proactive expansion of urology services, pathology capacity, and prostate cancer management infrastructure. Testicular cancer, a malignancy of young and middle-aged men, is rising comparatively rapidly. This further emerging trend warrants dedicated surveillance despite its small absolute burden.

Thyroid cancer warrants particular discussion given its complex epidemiological history [8]. Its trajectory (Figure 1) surged to a peak around 2012, when Korea briefly recorded the highest thyroid cancer incidence in the world. Incidence then declined partially after clinical guidelines were revised to discourage routine ultrasound screening of asymptomatic individuals. A subsequent re-ascent has occurred in recent years [8,17]. This pattern is the clearest illustration in our data of the interpretive caution required by data-quality considerations. The initial surge was driven overwhelmingly by ultrasound-based overdiagnosis of subclinical papillary microcarcinomas, rather than by a true increase in clinically significant disease. The projected near-plateau by 2030 is consistent with a post-overdiagnosis steady state.

A further important signal is the sustained, statistically significant increase across the hematological malignancies—multiple myeloma, Hodgkin and non-Hodgkin lymphoma, and leukemia (Results, Figure 5). These malignancies predominantly affect older adults. Their steady growth therefore mirrors Korea’s accelerating demographic aging. The share of the population aged ≥65 years has more than doubled since 2000 and is projected to exceed one-fifth before 2030. As this demographic expansion continues, hematological cancers will constitute an increasingly prominent component of the Korean oncological workload. This shift will require commensurate investment in hematology services, novel targeted therapies, and bone marrow transplantation capacity [21].

Placing these findings in an international context reinforces their interpretation. Global cancer statistics document a worldwide shift toward cancers of affluence—breast, prostate, colorectal, and corpus uteri—as countries progress along the socioeconomic development spectrum [22,23,24]. This shift is accompanied by declining infection-related cancers. Korea’s trajectory recapitulates this global pattern at an unusually compressed pace. Its cancer profile increasingly resembles that of high-income Western nations [25]. At the same time, Korea retains a comparatively high, though declining, stomach cancer burden. This persistence distinguishes it from most Western populations and reflects its distinctive infection-exposure history. We also compared the present 1999–2023 series with earlier Korean registry reports for 2009 and 2013 [12,26]. That comparison confirms that the transition documented here is a continuation and acceleration of trends already emerging a decade ago, rather than an artifact of the most recent reporting years.

Several limitations warrant acknowledgment. First, and most importantly, the study spans a 25-year period. Over this period, diagnostic technology, screening intensity, histopathological classification, and registry completeness all changed materially. Consequently, an unknown but non-negligible fraction of the observed increases—particularly for thyroid and prostate cancers—reflects improved detection and ascertainment rather than rising true incidence. The ecological design cannot disentangle these components quantitatively. Second, the ecological design precludes individual-level causal inference. It also cannot adjust for confounders such as smoking prevalence, obesity, alcohol use, or reproductive history. Third, ASIR values are those officially published by the KCCR. They depend on census-based population denominators, which may carry uncertainty for rare cancer types. Fourth, Holt–Winters forecasting assumes relative temporal stability in underlying trends. It cannot fully anticipate discontinuities arising from new screening guidelines, novel preventive interventions, or further pandemic-related disruptions. The widening 95% PIs with an increasing horizon appropriately reflect this uncertainty. Projections should therefore be read as scenario estimates rather than deterministic predictions. Fifth, the 2020 COVID-19 inflection introduces a structural break that the damped-trend parameter accommodates only partially. Finally, the analysis is restricted to the 24 major cancer types defined in national reporting, so rarer malignancies and finer histological subtypes are not individually examined.

## 5. Conclusions

Korean cancer epidemiology underwent a profound transformation between 1999 and 2023. The dominant pattern was a shift from infection-related malignancies toward hormonal, metabolic, and aging-related cancers. Female incidence rose by roughly two-thirds, whereas male incidence remained essentially flat. This divergence was driven predominantly by thyroid, breast, and corpus uteri cancers. Thyroid, prostate, and testicular cancers are the fastest-growing types by annual percent change. The hematological malignancies are also rising steadily with population aging. Total cancer incidence is projected to reach approximately 330,000 cases by 2030. The steepest relative growth is projected for prostate, kidney, and breast cancers. Part of the observed increase reflects concurrent improvements in diagnosis and case ascertainment. These trends should therefore be interpreted as a combination of genuine epidemiological transition and enhanced detection. Even so, these findings are cancer-type-resolved, sex-disaggregated, and grounded in 25 years of high-quality nationwide registry data. They provide an actionable evidence base for prospective capacity planning, specialist workforce development, and cancer screening program design in Korea.

## Figures and Tables

**Table 1:** Cancer-type-resolved incidence statistics for the 24 major cancer types in Korea (1999–2023): annual case counts, age-standardized incidence rates (ASIRs), sex-stratified 2023 burden, and annual percent change (APC).

Cancer Type (ICD-10)	Cases 1999	Cases 2023	ASIR 1999	ASIR 2023	Male 2023	Female 2023	ASIR Change (%)	APC (%)
Thyroid (C73)	3407	35,440	9.8	68.9	9326	26,114	+603.1	+7.56 ***
Lung (C33–C34)	13,224	32,953	59.8	57.5	21,846	11,107	−3.8	−0.13
Colorectum (C18–C20)	9783	32,610	40.8	58.7	19,156	13,454	+43.9	+1.01 *
Breast (C50)	5888	29,871	17.2	56.8	156	29,715	+230.2	+5.03 ***
Stomach (C16)	20,900	28,943	86.0	51.4	19,295	9648	−40.2	−2.20 ***
Other & unspecified (—)	7082	25,905	27.8	46.2	12,771	13,134	+66.2	+2.39 ***
Prostate (C61)	1454	22,640	7.4	39.2	22,640	0	+429.7	+6.98 ***
Liver (C22)	13,263	14,707	52.4	26.1	10,875	3832	−50.2	−2.90 ***
Pancreas (C25)	2614	9748	11.7	17.1	4925	4823	+46.2	+1.65 ***
Gallbladder & biliary tract (C23–C24)	3047	7997	14.0	13.8	4446	3551	−1.4	−0.05
Kidney (C64)	1416	7367	5.3	13.5	5073	2294	+154.7	+3.93 ***
Non-Hodgkin lymphoma (C82–C86, C96)	2064	6109	7.1	11.1	3490	2619	+56.3	+2.28 ***
Bladder (C67)	2195	5545	9.8	9.6	4465	1080	−2.0	−0.32 **
Lip, oral cavity & pharynx (C00–C14)	1741	4834	6.6	8.8	3469	1365	+33.3	+0.78 ***
Leukemia (C91–C95)	2168	4218	6.1	7.8	2444	1774	+27.9	+1.16 ***
Corpus uteri (C54)	728	4037	2.4	7.6	0	4037	+216.7	+5.03 ***
Ovary (C56)	1354	3299	4.5	6.2	0	3299	+37.8	+1.72 ***
Cervix uteri (C53)	4486	3144	14.2	5.9	0	3144	−58.5	−3.81 ***
Esophagus (C15)	1861	3142	8.2	5.5	2743	399	−32.9	−1.75 ***
Multiple myeloma (C90)	468	2099	1.9	3.7	1133	966	+94.7	+2.88 ***
Brain & CNS (C70–C72)	1359	2080	4.1	3.9	1145	935	−4.9	+0.01
Larynx (C32)	1101	1254	4.8	2.2	1178	76	−54.2	−3.18 ***
Hodgkin lymphoma (C81)	122	356	0.3	0.7	235	121	+133.3	+2.84 ***
Testis (C62)	129	315	0.2	0.6	315	0	+200.0	+5.39 ***

ASIR: age-standardized incidence rate per 100,000 (2020 Korean standard population). APC: annual percent change from log-linear regression on ASIR. Significance: *** *p* < 0.001; ** *p* < 0.01; * *p* < 0.05; no asterisk = not significant. ASIR Change (%): relative change in ASIR from 1999 to 2023. Male/Female 2023: sex-specific case counts. Rows are ordered by 2023 total case count.

**Table 2:** Holt–Winters incidence projections for all 24 major cancer types and total cancer in Korea (2023–2030).

Cancer Type	2023 (Obs.)	2024	2026	2028	2030	2023 → 2030 Change (%)
All cancers	288,613	294,845	307,091	318,875	330,213	+14.4
Thyroid	35,440	36,132	37,128	37,766	38,174	+7.7
Lung	32,953	34,102	35,932	37,744	39,537	+20.0
Colorectum	32,610	33,248	34,155	34,977	35,721	+9.5
Breast	29,871	31,583	34,155	36,702	39,223	+31.3
Stomach	28,943	29,130	29,286	29,414	29,520	+2.0
Other & unspecified	25,905	26,858	29,089	31,298	33,485	+29.3
Prostate	22,640	24,244	27,435	30,593	33,720	+48.9
Liver	14,707	14,544	14,295	14,096	13,938	−5.2
Pancreas	9748	10,173	10,968	11,754	12,533	+28.6
Gallbladder & biliary tract	7997	8185	8567	8945	9320	+16.5
Kidney	7367	7733	8447	9153	9852	+33.7
Non-Hodgkin lymphoma	6109	6433	6821	7206	7586	+24.2
Bladder	5545	5637	5939	6239	6536	+17.9
Lip, oral cavity & pharynx	4834	4971	5353	5732	6107	+26.3
Leukemia	4218	4241	4445	4648	4848	+14.9
Corpus uteri	4037	4222	4556	4886	5214	+29.2
Ovary	3299	3441	3607	3772	3936	+19.3
Cervix uteri	3144	3101	3004	2909	2816	−10.4
Esophagus	3142	3184	3355	3524	3691	+17.5
Multiple myeloma	2099	2172	2315	2456	2596	+23.7
Brain & CNS	2080	2165	2214	2261	2307	+10.9
Larynx	1254	1254	1266	1279	1292	+3.0
Hodgkin lymphoma	356	374	392	411	429	+20.5
Testis	315	357	375	392	409	+29.8

2023 values: observed KCCR data. 2024, 2026, 2028, and 2030: Holt–Winters damped exponential smoothing projections. Total cancer 95% prediction interval (PI) for 2030: 290,663–369,763 cases. Change (%): percentage change from 2023 observed to 2030 projected. Obs.: observed.

## Data Availability

The original data presented in the study are openly available in KOSIS at https://kosis.kr (accessed on 25 May 2026) (Table ID: DT_117N_A00025).
